# Medical Prospect of Melatonin in the Intervertebral Disc Degeneration through Inhibiting M1-Type Macrophage Polarization via SIRT1/Notch Signaling Pathway

**DOI:** 10.3390/biomedicines11061615

**Published:** 2023-06-01

**Authors:** Xinyu Dou, Qipeng Luo, Linzhen Xie, Xuchang Zhou, Chunyu Song, Meijuan Liu, Xiao Liu, Yunlong Ma, Xiaoguang Liu

**Affiliations:** 1 Department of Orthopedics, Peking University Third Hospital, Beijing 100191, China; douxinyupku@stu.pku.edu.cn (X.D.);; 2 Beijing Key Laboratory of Spinal Disease Research, Beijing 100191, China; 3 Engineering Research Center of Bone and Joint Precision Medicine, Ministry of Education, Beijing 100191, China; 4 Pain Medical Center, Peking University Third Hospital, Beijing 100191, China; 5 Peking University Fourth School of Clinical Medicine, Beijing 100035, China; 6 School of Sport Medicine and Rehabilitation, Beijing Sport University, Beijing 100084, China; 7 Department of Anesthesiology, Peking University Third Hospital, Beijing 100191, China; 8 Department of Endocrinology, Genetics and Metabolism, Beijing Children’s Hospital, Capital Medical University, National Center for Children’s Health, Beijing 100045, China

**Keywords:** melatonin, intervertebral disc degeneration, macrophage polarization, nucleus pulposus cell, SIRT1/Notch signaling pathway

## Abstract

The study aims to explore the medical prospect of melatonin (MLT) and the underlying therapeutic mechanism of MLT-mediated macrophage (Mφ) polarization on the function of nucleus pulposus (NP) in intervertebral disc degeneration (IDD). RAW 264.7 Mφs were induced by lipopolysaccharide (LPS) to simulate Mφ polarization and the inflammatory reaction of Mφs with or without MLT were detected. Conditioned medium (CM) collected from these activated Mφs with or without MLT treatment were further used to incubate NP cells. The oxidative stress, inflammation and extracellular matrix (ECM) metabolism in NP cells were determined. Then, the changes in SIRT1/Notch signaling were detected. The agonist (SRT1720) and inhibitor (EX527) of SIRT1 were used to further explore the association among MLT. The interaction between SIRT1 and NICD was detected by immunoprecipitation (IP). Finally, puncture-induced rat IDD models were established and IDD degrees were clarified by X-ray, MRI, H&E staining and immunofluorescence (IF). The results of flow cytometry and inflammation detection indicated that LPS could induce M1-type Mφ polarization with pro-inflammatory properties. MLT significantly inhibited the aforementioned process and inhibited M1-type Mφ polarization, accompanied by the alleviation of inflammation. Compared with those without MLT, the levels of oxidative stress, pro-inflammatory cytokines and ECM catabolism in NP cells exposed to CM with MLT were markedly downregulated in a dose-dependent manner. The inhibition of SIRT1 and the enhancement of Notch were observed in activated Mφs and they can be reversed after MLT treatment. This prediction was further confirmed by using the SRT1720 and EX527 to activate or inhibit the signaling. The interaction between SIRT1 and NICD was verified by IP. In vivo study, the results of MRI, Pfirrmann grade scores and H&E staining demonstrated the degree of disc degeneration was significantly lower in the MLT-treated groups when compared with the IDD control group. The IF data showed M1-type Mφ polarization decreased after MLT treatment. MLT could inhibit M1-type Mφ polarization and ameliorate the NP cell injury caused by inflammation in vitro and vivo, which is of great significance for the remission of IDD. The SIRT1/Notch signaling pathway is a promising target for MLT to mediate Mφ polarization.

## 1. Introduction

Intervertebral disc degeneration (IDD), as a trigger for most spinal disorders such as disc herniation, spondylosis and lumbar spinal stenosis [1], has been considered as the major cause of low back pain (LBP) [2]. The latter causes the disability and pain of up to 637 million people worldwide [3]. Therefore, LBP incurs great costs for society, being responsible for >30% of absences from work, causing increasing healthcare costs and considerable loss of productive force [4]. In the early stage of IDD, a great deal of immunocytes, especially macrophages (Mφs), infiltrated the closed nucleus pulposus (NP) due to the rupture of the annulus fibrosus (AF) and further induced the chemotaxis of the inflammation-related or pain-related factors in the intervertebral disc (IVD) through direct phagocytosis or indirect mediating of neuroimmune mechanism.

Mφ itself is highly plastic, and its type will change dynamically with external stimuli [5]. Mφ can exhibit the M2 type in a non-inflammatory state but can rapidly polarize to the M1 type with the stimulation of inflammation [6]. In fact, the cells in the degenerated IVD can also secrete inflammatory mediators and activate Mφs. Co-culture of the activated Mφs with IVD cells can significantly upregulate the levels of pro-inflammatory factors such as interleukins (ILs) and tumor necrosis factor-α (TNF-α) [7,8]. The deterioration of inflammation caused by the M1-type Mφs could further evoke a series of changes, such as excessive oxidative stress, autophagy, and apoptosis in IVD cells. It ultimately damaged the metabolic balance of the extracellular matrix (ECM) and aggravates the development of IDD.

Melatonin (MLT), an indoleamine hormone mainly produced in the pineal gland, is widely studied for regulating sleep and circadian rhythms. Recently, it is also considered as an important mediator in various pathophysiological processes such as anti-apoptotic activity, anti-inflammatory activity, modulation of the permeability transition pore, modulation of autophagy, analgesic process and so on [9]. It functions primarily by binding to cell membrane receptors 1 and 2 (MT1 and MT2) [5] or nuclear receptors including the nuclear receptor retinoid Z receptor (RZR)/retinoid acid receptor (ROR) [10]. Multiple studies demonstrated that MLT can induce the M2 polarization of Mφ in damaged tissues and downregulate the levels of the inflammatory factors and oxidative stress molecules, thereby exerting anti-inflammation [11] and antioxidant effects [12,13]. The internal correlation between MLT and IDD was first reported by Turgut et al. in 2003, with an evidence of the IDD phenotype after pineal resection in Hybro Broiler chickens and the negative correlation between the severity of IDD and the level of MLT [14]. Subsequently, an increasing number of studies have reported that the application of MLT could restore the degenerated IVD to a close to normal state. The potential regulatory mechanisms include alleviating oxidative stress, regulating autophagy [15] and apoptosis [16], and improving the metabolic balance of ECM in IVD. 

Sirtuin 1 (SIRT1), as a highly conserved NAD^+^-dependent histone deacetylase, is considered to be an important regulator of inflammation [17]. Its high expression can inhibit the activity of a variety of inflammatory transcription factors (such as NF-κB and FOXO) and the process of oxidative stress, leading to the inhibition of inflammation. Recently, new insights have been emerging concerning SIRT1 as a secondary mediator of the anti-inflammatory effects of MLT. SIRT1 was not only shown to be upregulated by melatonin in experimental cell cultures and animal models [18,19]; MLT effects were also shown to be diminished in *SIRT1*^−/−^ mice [20] or attenuated by SIRT1 inhibitors, such as EX527 or sirtinol or knockdown by *SIRT1* siRNA [21,22,23]. In several studies, SIRT1 has been shown to exert its anti-inflammation action by inhibiting Notch signaling. Notch signaling is normally activated by the engagement of Notch receptors by Notch ligands expressed on adjacent cells. Notch protein is released from the intracellular domain of Notch (NICD) into cytoplasm after three times shearing, then enters the nucleus and binds with the transcription factor (CBF-1/Suppressor of hairless/Lag, CSL) to form the NICD/CSL transcriptional activation complex, which drives the expression of downstream target genes such as the genes of family bHLH transcription factor 1 (HES1), Hes-related family BHLH transcription factor YRPW motif 2 (HEY2) and Notch-regulated ankyrin repeat protein (NRARP), and thereby executes the functions of Notch [24]. SIRT1 inhibited Notch signaling through NICD deacetylation [25], whereas *SIRT1* knockdown by siRNA increased the levels of Notch1 protein and mRNA expression [26]. It has been reported that Notch signaling has a specific proinflammatory effect in Mφs. Notch receptor, ligand and target gene expression in Mφs can be further enhanced in response to pro-inflammatory stimuli [27]. Since SIRT1 is a secondary mediator of the effects of MLT, SIRT1 could inhibit Notch signaling, which plays an important role in Mφ polarization and inflammatory responses. It would be of interest to study whether the effects of MLT on inhibiting M1 polarization of Mφs and its inflammatory responses were mediated by SIRT1/Notch signaling.

However, whether the regulation of Mφ polarization contributes to the therapeutic capability of MLT in IDD has not been identified so far. To determine the clinical translational potential of MLT for the treatment of IDD, the Mφ M1 polarization conditioned medium (CM) from the lipopolysaccharide (LPS)-stimulated RAW 264.7 Mφs was utilized to induce the injury of primary mouse NP cells and the puncture rat model of IDD was established. Current mechanism studies demonstrated that MLT could ameliorate IDD through the inhibition of M1 polarization of Mφs via the SIRT1/Notch signaling pathway, providing the novel evidence to utilize the clinical management of IDD with MLT.

## 2. Materials and Methods

### 2.1. Isolation and Primary Culture of Mouse NP Cells

IVDs were harvested from the lumbar spines of C57BL/6. The NP tissue was carefully separated from the annulus fibrosus by a stereotaxic microscope. The NP tissues were then cut into small fragments and digested for 1 h in 0.25% type II collagenase (Sigma, St. Louis, MO, USA) and for 5 min in trypsin-EDTA solution (0.2% trypsin, 1 mM EDTA, Gibco, Grand Island, NY, USA) at 37 °C, respectively. After isolation, NP cells were cultured in Dulbecco’s modified Eagle medium (DMEM, HyClone, Thermo Scientific, Logan, UT, USA) containing 20% fetal bovine serum (FBS, Gibco, Grand Island, NY, USA) under conventional incubation conditions (37 °C and 5% CO_2_). NP cells were passaged by using trypsin-EDTA solution (0.25% trypsin, 1 mM EDTA, Gibco, Grand Island, NY, USA) when they had 80–90% confluency. NP cells in passage P3 were used for subsequent experiments.

### 2.2. Culture of Mouse RAW 264.7 Mφs

The mouse Mφ cell line RAW 264.7 was obtained from the National Experimental Cell Resource Sharing platform (Beijing, China) and cultured in DMEM (HyClone, Thermo Scientific, Logan, UT, USA) supplemented with 10% FBS (Gibco, Grand Island, NY, USA) at 37 °C in a humidified atmosphere containing 5% CO_2_.

### 2.3. Preparation of CM

The CM of RAW 264.7 Mφs was collected as follows: (1) 3 × 10^6^ RAW 264.7 Mφs were seeded in 10 cm culture dishes and cultured with LPS (1 μg/mL) treatment for 6 h. (2) RAW264.7 Mφs were washed three times with Phosphate Buffered Saline (PBS) (HyClone, Thermo Scientific, Logan, UT, USA) and re-cultured in the normal completed medium with MLT for 24 h. (3) RAW264.7 Mφs were washed three times with PBS (HyClone, Thermo Scientific, Logan, UT, USA) and re-cultured in the normal completed medium for another 24 h. (4) CM was collected and centrifuged briefly to remove cell debris, and the remaining supernatant was considered as CM and was used for subsequent experiments.

### 2.4. Rat Model of IDD

Twelve-week-old male SpragueeDawley rats (*n* = 30), used for this study (Peking university third hospital, Beijing, China), were randomly divided into three groups (IDD control group, IDD + 5 mg/kg-MLT group and IDD + 10 mg/kg-MLT group, *n* = 10). Then, the rats were anesthetized by 2% (*w*/*v*) pentobarbital (40 mg/kg). After locating the caudal disc (Co7 and Co8) through X-ray, the whole layer of AF to the NP tissue was vertically punctured through the skin using the 18 G needles. The puncture depth was controlled at 6 mm, and the needle was rotated 360° and kept in the position for 2 min. Thereafter, the rats were monitored daily to observe their health condition. The follow-up drugs were administered by intraperitoneal injection.

### 2.5. Reagents and Antibodies

MLT was obtained from Rhawn (Shanghai, China). SIRT1 agonist SRT1720, SIRT1 inhibitor EX527 and LPS were acquired from MedChemExpress (Shanghai, China). The following primary polyclonal antibodies were utilized: rabbit anti-mouse IL-4 (1:500, Cat. No. PAA077Mu01), rabbit anti-mouse IL-6 (1:500, Cat. No. PAA079Mu01), rabbit anti-mouse IL-10 (1:500, Cat. No. PAA056Mu01), rabbit anti-mouse TNF-α (1:500, Cat. No. PAA133Mu01), rabbit anti-mouse inducible nitric oxide synthase (iNOS) (1:500, Cat. No. PAA837Mu01), rabbit anti-mouse NICD (1:500, Cat. No. PAG797Mu01), rabbit anti-mouse cyclooxygenase-2 (COX-2) (1:500, Cat. No. PAD666Mu01), rabbit anti-mouse collagen type II α 1 (COL2A1) (1:500, Cat. No. PAD194Mu01), rabbit anti-mouse metalloproteinase-1 (TIMP-1) (1:500, Cat. No. PAA552Mu01), rabbit anti-mouse matrix metalloproteinase 13 (MMP-13) (1:500, Cat. No. PAA099Mu01), rabbit anti-mouse HES1 (1:500, Cat. No. PAK289Hu01) and rabbit anti-mouse NRARP (1:500, Cat. No. PAS396Mu01) were obtained from Cloud-Clone Corp (Wuhan, China). The rabbit anti-mouse antibodies of a disintegrin and ADAM metallopeptidase with thrombospondin type 1 motif 4 (ADAMTS4) (1:500, Cat. No. DF6986), ADAMTS5 (1:500, Cat. No. DF13268), Notch 1 (1:500, Cat. No. AF5307), HEY2 (1:500, Cat. No. AF9092) were obtained from Affinity Biosciences (Zhenjiang, China). The rabbit anti-mouse monoclonal CD86 antibody (1:300, Cat. No. ab275357), the rabbit anti-mouse monoclonal acetyl-lysine antibody (1:500, Cat. No. ab190479) and the mouse anti-mouse monoclonal SIRT1 antibody (1:500, Cat. No. ab110304) were acquired from Abcam (Cambridge, MA, USA). The rabbit anti-rat polyclonal CD68 antibody (1:100, Cat. No. 28058-1-AP) and the rabbit anti-rat polyclonal CD206 antibody (1:100, Cat. No. 18704-1-AP) were acquired from Proteintech (Rosemont, IL, USA). The fluorescein isothiocyanate (FITC)-labeled goat anti-rabbit secondary antibody (1:100, Cat. No. SF134) and horseradish peroxidase-labeled goat anti-rabbit secondary antibody (1:100, Cat. No. SE134) were ordered from Solarbio (Beijing, China). The 4′,6-diamidino-2-phenylindole (DAPI) (Cat. No. C1006) was acquired from Beyotime (Shanghai, China). 

### 2.6. MTT Assay 

Cytotoxicity was evaluated by MTT assay (Sigma-Aldrich, St. Louis, MO, USA). RAW264.7 Mφs were plated in 96-well plates at a density of 6 × 10^3^ cells/well and incubated at 37 °C for 24 h. MLT was dissolved in DMSO and the cells were treated with 0.01 μM, 0.1 μM and 1 μM concentrations of MLT. Cells treated with only 0.1% DMSO served as a negative control. After incubation for 24 h in a humidified incubator at 37 °C, 10 μL of MTT solution was added to each well and incubated at 37 °C for 4 h. After careful removal of the medium, 200 μL DMSO solution was added to each well. Then, the plates were gently shaken at room temperature for 10 min. The absorbance was read at the wavelength of 490 nm using a microplate reader (Thermo Scientific, Vario Skan Flash, Waltham, MA, USA). Five replicate wells were used for each concentration and each experiment was repeated three times. Cell viability (%) = (OD (treatment) − OD (blank))/(OD (control) − OD (blank)) × 100%.

### 2.7. Flow Cytometry Assay

The phenotypic characterizations of M1-type and M2-type Mφs were performed using flow cytometry. Cells (2 × 10^5^ cells) were washed twice and resuspended in 100 μL PBS and incubated with respective fluorochrome-labeled antibodies and isotype controls for 30 min, protected from light. The characterization of the M2 type was performed using CD206, whereas the M1 type was investigated using CD86 antibodies. Flow cytometry was carried out on a Becton–Dickinson FACSCalibur (BD Biosciences, Franklin Lakes, NJ, USA). The percentage of M1/M2 was calculated.

### 2.8. Reactive Oxygen Species (ROS), Malondialdehyde (MDA), and Glutathione (GSH) Assays

Intracellular ROS was detected by a ROS assay kit (Beyotime, Shanghai, China). Cells were incubated with 10 µM/L 2′,7′-dichlorofluorescein diacetate (DCFH-DA) at 37 °C for 20 min and then washed with serum-free medium three times. DCFH is a cell impermeable agent that can be oxidized through the reaction with ROS into DCF, a compound that emits green fluorescence. Its mean fluorescence intensity positively related to the level of intracellular ROS, which was determined with a Becton–Dickinson FACSCalibur (BD Biosciences).

The generation of MDA was determined using a commercial kit (Beyotime, Shanghai, China) according to the manufacturer’s protocol. Briefly, the harvested cells were lysed at 4 °C for 60 min, and the supernatant was collected after centrifugation at 12,000× *g* for 10 min. Protein concentration in the supernatant was detected using a bicinchoninic acid (BCA) kit (Beyotime, Shanghai, China). In addition, 100 µL supernatant was placed into a centrifuge tube and 200 µL MDA testing solution was added. The mixture was boiled for 15 min and centrifugated at 1000× *g* for 10 min. Finally, 200 µL prepared supernatant was loaded into a 96-well plate to read absorbance at 532 nm using a microplate reader (Rayto, Shenzhen, China). 

GSH was detected with the GSH detection kit (Beyotime, Shanghai, China) following the manufacturer’s instructions. Briefly, the harvested cells were washed with PBS and mixed with 30 µL protein removal reagent M, and then frozen and thawed twice using liquid nitrogen and 37 °C water. The supernatant was collected after centrifugation at 10,000× *g* for 10 min at 4 °C and used for GSH detection. GSH content was detected with a microplate reader (Rayto, Shenzhen, China) at a wavelength of 412 nm.

### 2.9. Real-Time Quantitative Polymerase Chain Reaction (RT-qPCR)

Total RNA was extracted from cells using Trizol reagent (TransGen Biotech, Beijing, China) according to the manufacturer’s protocols. cDNA was synthesized from 1 μg of total RNA by using the Quantscript RT Kit (Beyotime, Shanghai, China) according to the supplier’s protocol. cDNA was amplified using a TransStart^®^ Top Green qPCR SuperMix kit (TransGen Biotech, Beijing, China) on the Eppendorf Realplex4 instrument (Eppendorf, Hamburg, Germany). The reaction conditions were as follows: 95 °C for 3 min, followed by 40 cycles at 95 °C for 30 s and at 55 °C for 20 s, and extension for 20 s at 72 °C. The sequences of primers were presented in Table 1. The 2^−ΔΔCt^ method was used to calculate the relative RNA expression levels of target genes.

### 2.10. Western Blot

Cells were lysed with RIPA lysis buffer (Beyotime, Shanghai, China) on ice for 60 min and cell lysates were collected by centrifugation at 12,000× *g* for 15 min at 4 °C. Protein concentration in cell extracts was determined using the BCA method (Beyotime, Shanghai, China). Protein samples (100 µg each sample per lane) were separated by 10% SDS-PAGE and transferred to a polyvinylidene difluoride (PVDF) membrane (Millipore, Schwalbach, Germany). The membrane was then blocked with 3% bull serum albumin (BSA) at room temperature for 2 h and incubated with the primary antibodies at 4 °C overnight. Then, the prepared membranes were washed six times with tris buffered saline tween (TBST). Following washing, the membrane was incubated with corresponding secondary antibodies at 37 °C for 2 h. Finally, the blots were detected by an enhanced chemiluminescence kit (Beyotime, Shanghai, China). Signal intensity was quantified with ImageJ software version 1.8.0 with β-actin used as an internal control. 

### 2.11. Immunoprecipitation (IP) Assay

Protein samples for the IP assay were prepared as stated above, and the concentrations in cell extracts were determined using the BCA method (Beyotime, Shanghai, China). For IP, 1 mg of protein sample was incubated with acetyl-lysine, NICD antibody (1:1000), and control IgG at 4 °C overnight. Subsequently, the cell lysates were incubated with 100 µL Protein A/G Agarose (Beyotime, Shanghai, China) at 4 °C for 4 h before being centrifuged. Afterward, the supernatants were discarded, and the pellets were resuspended with 100 µL SDS sample buffer. The suspension was boiled at 100 °C for 5 min and collected for Western bolt.

### 2.12. X-ray Radiograph and Magnetic Resonance Imaging (MRI)

X-ray radiograph was respectively performed for rat models of IDD at 3 weeks and 6 weeks after surgery to preliminarily evaluate the degree of IVD degeneration by the digital X-ray system (United-imaging, Shanghai, China); furthermore, to more clearly detect the signal intensity changes of IVDs of rats and evaluate the condition about the degeneration of IVD. Coronal and sagittal MRI was respectively performed for rat models of IDD at 3 weeks and 6 weeks after surgery using the MRI system (United-imaging, China). According to the results of T2-weighted images, the target degenerative IVDs were graded by the Pfirrmann grade system to quantify the degeneration degree of IVDs [28].

### 2.13. Histopathologic Analysis

The rat IDD models were sacrificed at 3 and 6 weeks after surgery. The IVDs of Co7 and Co8 were obtained and fixed in 4% paraformaldehyde. After the decalcification using EDTA decalcifying solution, the samples were dehydrated and embedded in paraffin for serial sectioning. Then, the sections were stained by hematoxylin-eosin (H&E) staining to evaluate the morphological changes of NP cells.

### 2.14. Immunofluorescence

Treated rat NP cells planked on coverslips were fixed with 4% paraformaldehyde for 30 min and then washed by PBS. After incubating with 0.1% Triton X-100 for 5 min, samples were blocked by 10% goat serum for 1 h at 37 °C. Then, samples were incubated with primary antibodies such as CD68 (1:100) and CD206 (1:100) at 4 °C overnight. Then, samples were washed with PBS and subsequently incubated with Alexa Fluor488 or Alexa Fluor594 conjugated secondary antibodies for 1 h at 37 °C and stained nucleus by DAPI. The coverslips were then observed by a confocal microscope (Zeiss, Germany). 

### 2.15. Statistical Analysis

Data were presented as mean ± standard deviation (SD). Comparison of continuous data between the two groups was performed by independent Student’s *t*-test, which was analyzed by using IBM SPSS software version 20 (IBM Corp., Armonk, NY, USA). Differences at *p*-value  <  0.05 were considered statistically significant.

## 3. Results

### 3.1. Low Concentration MLT Displays No Cytotoxicity on RAW 264.7 Mφs

**Figure 1:**
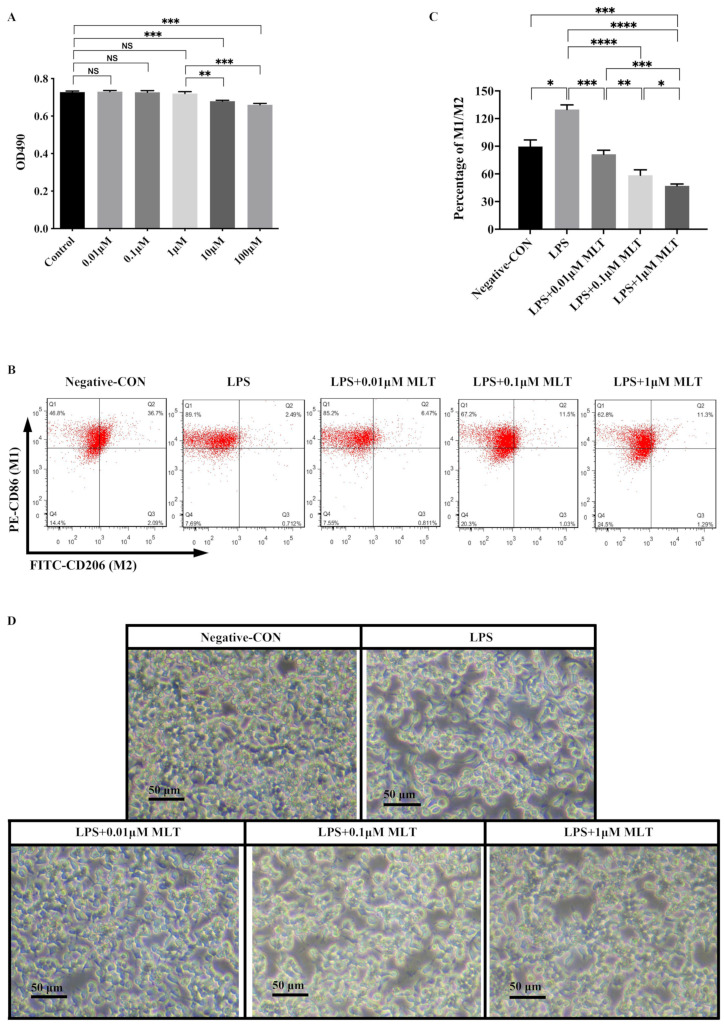
Inhibition effect of MLT on M1-type polarization in Mφs. RAW 264.7 Mφs were treated with LPS 6 h and followed by the presence or absence of MLT for 24 h, then followed by different testing. (**A**) MLT displayed very little cytotoxicity effects in Mφs at different concentrations of MLT, ranged from 0.01 up to 1 μM, while it displayed significant cytotoxicity at concentrations ≥ 10 μM (*n* = 3). (**B**,**C**) Reduction of the ratio of M1/M2-type Mφs by MLT in Mφs measured by flow cytometry assay. (**D**) Observation of morphological characteristics in Mφs. Data are expressed as mean ± standard deviation. The two groups among the six or five groups are compared by using an independent *t*-test. * *p* < 0.05, ** *p* < 0.01, *** *p* < 0.001, **** *p* < 0.0001.

To evaluate the in vitro cytotoxicity of MLT, RAW 264.7 Mφs was treated with different gradient concentrations of MLT (0.01 μM, 0.1 μM, 1 μM, 10 μM and 100 μM), and then cell viability was examined using MTT assays. As shown in Figure 1A, MLT treatment resulted in significant cytotoxicity at concentrations ≥ 10 μM, while it showed very little cytotoxicity at low concentrations (below 10 μM). Thus, 0.01 μM, 0.1 μM and 1 μM concentrations of MLT were used in the following experiments.

### 3.2. Inhibition of M1-Type Polarization by MLT in Mφs

**Figure 2:**
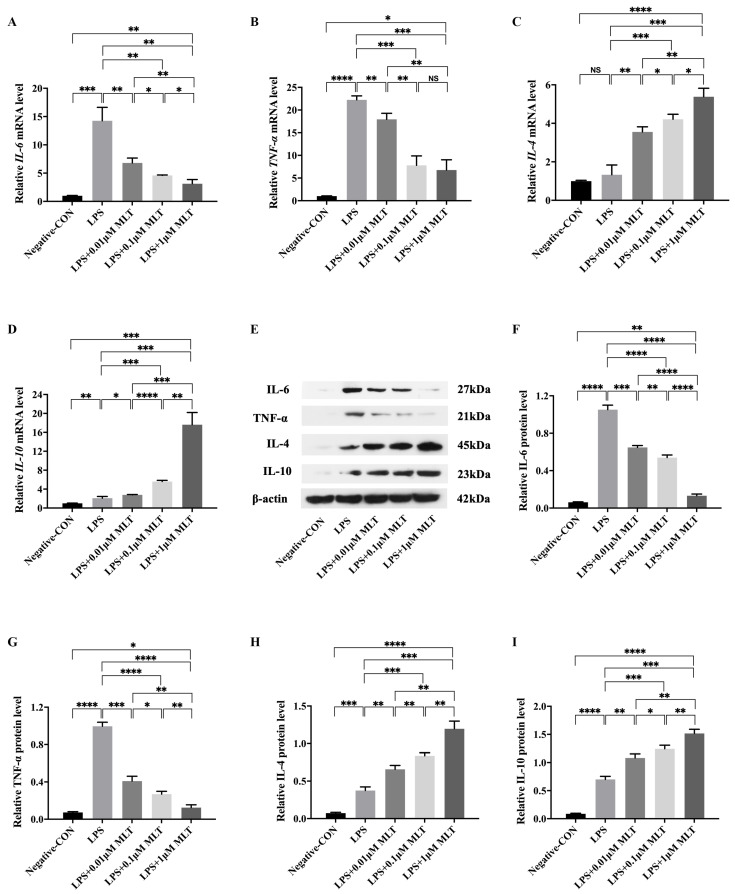
Downregulated M1-type Mφ markers and upregulated M2-type Mφ markers of Mφs by the treatment of MLT. RAW 264.7 Mφs were treated with LPS 6 h and followed by the presence or absence of MLT for 24 h, then followed by RT-qPCR and Western blot. (**A**–**D**) The relative mRNA levels of M1-type Mφ genes (*IL-6* and *TNF-α*) and M2-type Mφ genes (*IL-4* and *IL-10*) were measured by RT-qPCR analyses (*n* = 3). (**A**) The levels of *IL-6* mRNA were reduced in the presence of MLT treatment at different dosages. (**B**) The levels of *TNF-α* mRNA were decreased in the presence of MLT treatment at different dosages. (**C**) The levels of *IL-4* mRNA were increased in the presence of MLT treatment at different dosages. (**D**) The levels of *IL-10* mRNA were enhanced in the presence of MLT treatment at different dosages. (**E**–**I**) The relative protein levels of Mφ markers in Mφs were measured by Western blot analyses (*n* = 3). (**E**) Western blotting showed the downregulation of M1-type Mφ markers and upregulation of M2-type Mφ markers. (**F**) Quantitative analysis showed the reduced levels of IL-6 protein after the treatment with MLT at different dosages. (**G**) Quantitative analysis showed the reduced levels of TNF-α protein after the treatment with MLT at different dosages. (**H**) Quantitative analysis showed the enhanced levels of IL-4 protein after the treatment with MLT at different dosages. (**I**) Quantitative analysis showed the increased levels of IL-10 protein after the treatment with MLT at different dosages. Data are expressed as mean ± standard deviation. The two groups among the five groups are compared by using an independent *t*-test. * *p* < 0.05, ** *p* < 0.01, *** *p* < 0.001, **** *p* < 0.0001.

To investigate whether MLT could regulate the type switch and the expression of inflammatory cytokines in macrophages after LPS stimulation, RAW 264.7 Mφs were treated with LPS (1 μg/mL) first. Flow cytometry analysis found that the percentage of M1-type Mφs increased, while that of M2-type Mφs decreased in RAW 264.7 Mφs exposed to LPS when compared to the negative control group (Figure 1B). To quantify this observed phenomenon, the ratio of M1-type to M2-type Mφs (M1/M2) was calculated, and a higher M1/M2 ratio in the LPS-treated group was observed than that of the negative control (Figure 1C). As shown in Figure 1D, when the Mφs were cultured in the negative control medium, the majority of cells were observed to exhibit a circular morphology, while the morphology of the cells cultured in the presence of LPS were altered. These data indicated that LPS could promote the polarization of the Mφs towards M1 type. After LPS stimulation, the mRNA levels of M1-type Mφ markers, including *IL-6* and *TNF-α*, were markedly increased (Figure 2A,B). These results were consistent with those obtained from Western blot (Figure 2E–G).

Next, to investigate whether MLT could partly ameliorate the above phenomena. RAW 264.7 Mφs were treated with LPS (1 μg/mL), simultaneously with or without MLT (0.01 μM, 0.1 μM and 1 μM) for 24 h and the CM from treated RAW 264.7 Mφs was prepared. Flow cytometry analysis showed that after treatment with MLT, the M1/M2 ratio was lower in a dose-dependent manner when compared with those in LPS-treated alone Mφs. (Figure 1B,C). The morphological alterations related to Mφ activation were observed when Mφs were treated with LPS and MLT (Figure 1D). In addition, both RT-qPCR and Western blot revealed that M1-type Mφ markers, including IL-6 and TNF-α, were significantly downregulated, while M2-type Mφ markers, including IL-4 and IL-10, were all upregulated in the LPS + MLT-treated Mφs when compared with those in LPS-treated alone Mφs (Figure 2A–I). These data demonstrated that LPS treatment could promote M1-type polarization of Mφs and expression of pro-inflammatory cytokines, which could be inhibited by MLT to some extent.

### 3.3. Induction of Cell Injury in NP Cells by M1-Type Mφ Polarization

**Figure 3:**
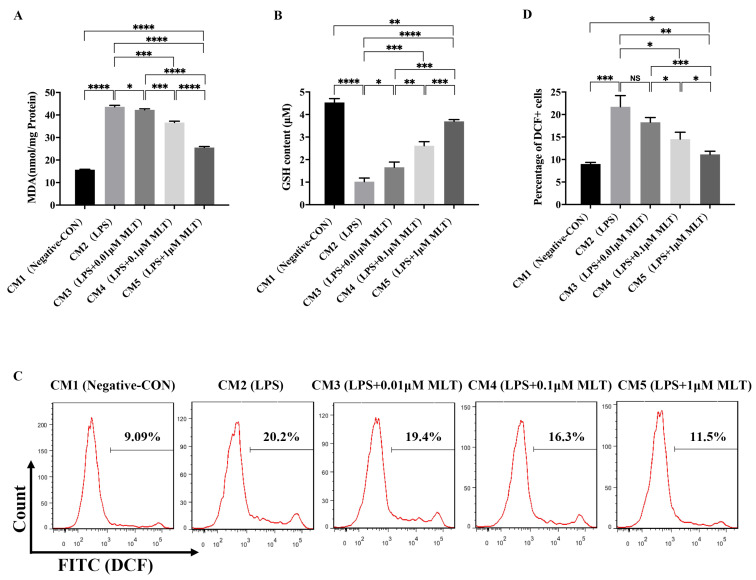
Protective effects of MLT against cell injury in NP cells induced by M1-type polarization of Mφ. NP cells were treated with different CMs collected from the LPS-stimulated RAW 264.7 Mφs in the presence or absence of MLT for 24 h, then followed by different analysis. (**A**–**D**) Reduction of oxidative stress by MLT in NP cells. (**A**) MLT decreased the level of MDA. (**B**) MLT increased the level of GSH. (**C**,**D**) The ROS levels were declined after treatment with different concentrations of MLT. The levels of GSH and MDA in NP cells were determined by ELISA (*n* = 3). The intracellular ROS levels in NP cells were measured by flow cytometry assay. Data are expressed as mean ± standard deviation. The two groups among the five groups are compared by using an independent *t*-test. * *p* < 0.05, ** *p* < 0.01, *** *p* < 0.001, **** *p* < 0.0001.

**Figure 4:**
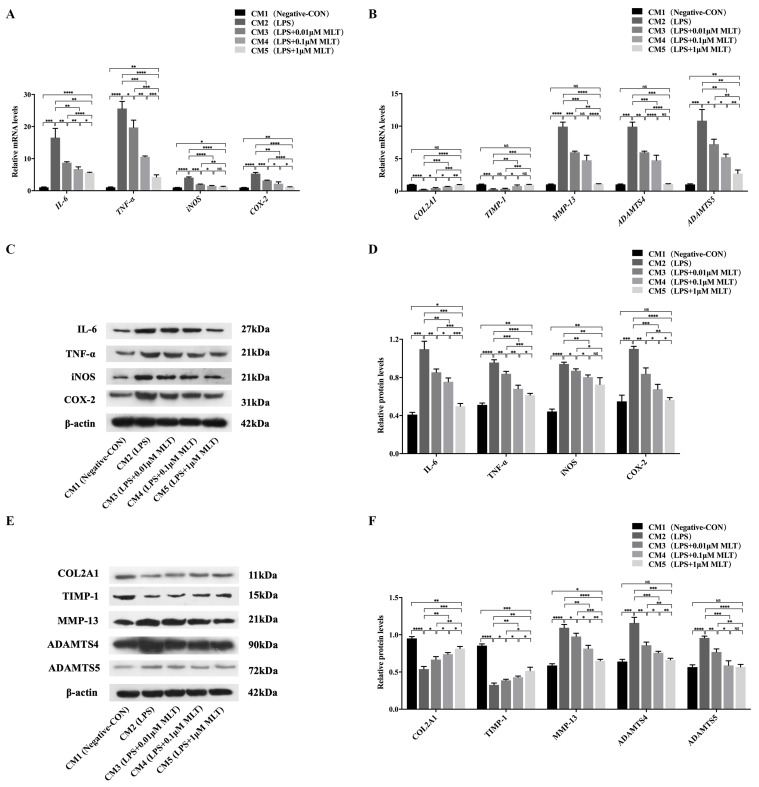
Protection on NP cells against the CM-induced inflammation and imbalanced ECM homeostasis by the treatment of MLT. NP cells were treated with different CMs collected from the LPS-stimulated RAW 264.7 Mφs in the presence or absence of MLT for 24 h, then followed by different analysis. (**A**) The relative mRNA levels of pro-inflammation cytokines in NP cells were measured by RT-qPCR analyses (*n* = 3). The levels of *IL-6*, *TNF-α*, *iNOS* and *COX-2* mRNA declined in the presence of MLT treatment to Mφs at different dosages. (**B**) The relative mRNA levels of matrix anabolic and degrading enzymes in NP cells were measured by RT-qPCR analyses (*n* = 3). The levels of *COL2A1* and *TIMP-1* mRNA were upregulated, while the levels of *MMP-13*, *ADAMTS4* and *ADAMTS5* mRNA were downregulated in the presence of MLT treatment to Mφs at different dosages. (**C**,**D**) The relative protein levels of pro-inflammation cytokines in NP cells were measured by Western blot analyses (*n* = 3). (**C**) Western blotting showed the decline of inflammation-associated proteins. (**D**) Quantitative analysis showed the reduced levels of IL-6, TNF-α, iNOS and COX-2 protein after treatment with MLT to Mφs at different dosages. (**E**,**F**) The relative protein levels of matrix anabolic and degrading enzymes in NP cells were measured by Western blot analyses (*n* = 3). (**E**) Western blotting showed the different changes of ECM protein productions in NP cells. (**F**) Quantitative analysis showed the increased levels of COL2A1 and TIMP-1 protein, and the reduced levels of MMP-13, ADAMTS4 and ADAMTS5 protein after the treatment with MLT to Mφs at different dosages. Data are expressed as mean ± standard deviation. The two groups among the five groups are compared by using an independent *t*-test. * *p* < 0.05, ** *p* < 0.01, *** *p* < 0.001, **** *p* < 0.0001.

To identify the effects of M1-type polarization of Mφs on NP cells, NP cells were cultured in the series of CMs obtained by drug-stimulated RAW 264.7 Mφs (CM1: Negative control Mφs; CM2: LPS-treated Mφs; CM3: LPS + 0.01 μM MLT- treated Mφs; CM4: LPS + 0.1 μM MLT-treated Mφs; CM5: LPS + 1 μM MLT-treated Mφs) for 24 h. As shown in Figure 3, the NP cells cultured with CM2 had a significant decrease in the levels of antioxidant enzyme GSH (Figure 3B), while MDA (Figure 3A) and intracellular ROS levels (Figure 3C,D) increased relative to that of the CM1-treated control group. These data indicated that M1-type polarization of Mφs promoted the oxidative stress response in NP cells. In addition, the RT-qPCR results showed that compared with the CM1-treated NP cells, mRNA levels of pro-inflammation-related genes, including *IL-6*, *TNF-α*, *iNOS* and *COX-2* were all markedly upregulated in the CM2-treated group (Figure 4A). These results were consistent with those obtained from Western blot (Figure 4C,D), which further indicated that M1-type polarization of Mφs evoked the inflammatory reactions in NP cells. Furthermore, the effects of M1-type polarization of Mφs on the synthesis and degradation of ECM in NP cells were also explored. Both RT-qPCR and Western blot demonstrated that matrix anabolic markers, including COL2A1 and TIMP-1, were markedly decreased, whereas matrix degradation markers, including MMP-13, ADAMTS4 and ADAMTS5, were significantly increased in the CM2-treated NP cells in comparison with CM1-treated NP cells (Figure 4B,E,F). These results confirmed that the homeostasis of ECM in NP cells were disturbed by M1-type polarization of Mφs.

### 3.4. Protection on NP Cells against Cell Injury by the Treatment with MLT via Inhibiting M1-Type Mφ Polarization 

To further explore whether inhibition of M1-type polarization of Mφs by MLT treatment may ameliorate the above cell injury in NP cells, the following experiments were carried out to demonstrate it. Firstly, treatment with MLT (CM3-, CM4- and CM5-treated groups) significantly decreased levels of MDA (Figure 3A) and intracellular ROS levels (Figure 3C,D) while markedly increasing content of GSH (Figure 3B) in NP cells, in a dose-dependent manner, relative to those of the MLT-untreated group (CM2-treated group). Secondly, RT-qPCR showed that compared with the MLT-untreated group, mRNA levels of pro-inflammation-related genes, including *IL-6*, *TNF-α*, *iNOS* and *COX-2*, were all markedly downregulated in the MLT-treated groups (CM3-, CM4- and CM5-treated groups) in a dose-dependent manner (Figure 4A). Consistent with RT-qPCR results, Western blot further revealed that protein levels of these pro-inflammation cytokines in MLT-treated NP cells also significantly declined in comparison with those in the MLT-untreated group (Figure 4C,D). In addition, both RT-qPCR and Western blot results showed that ECM anabolic markers, including COL2A1 and TIMP-1, were significantly upregulated, while ECM degradation markers, including MMP-13, ADAMTS4 and ADAMTS5, were all downregulated in the MLT-treated NP cells when compared with those in the MLT-untreated NP cells (Figure 4B,E,F). These data indicated that treatment with MLT could resist cell injury induced by M1-type polarization of Mφs in NP cells.

### 3.5. Regulation of SIRT1/Notch Signal Pathway Contributes to the Effect of MLT on Inhibiting M1-Type Polarization of Mφs

**Figure 5:**
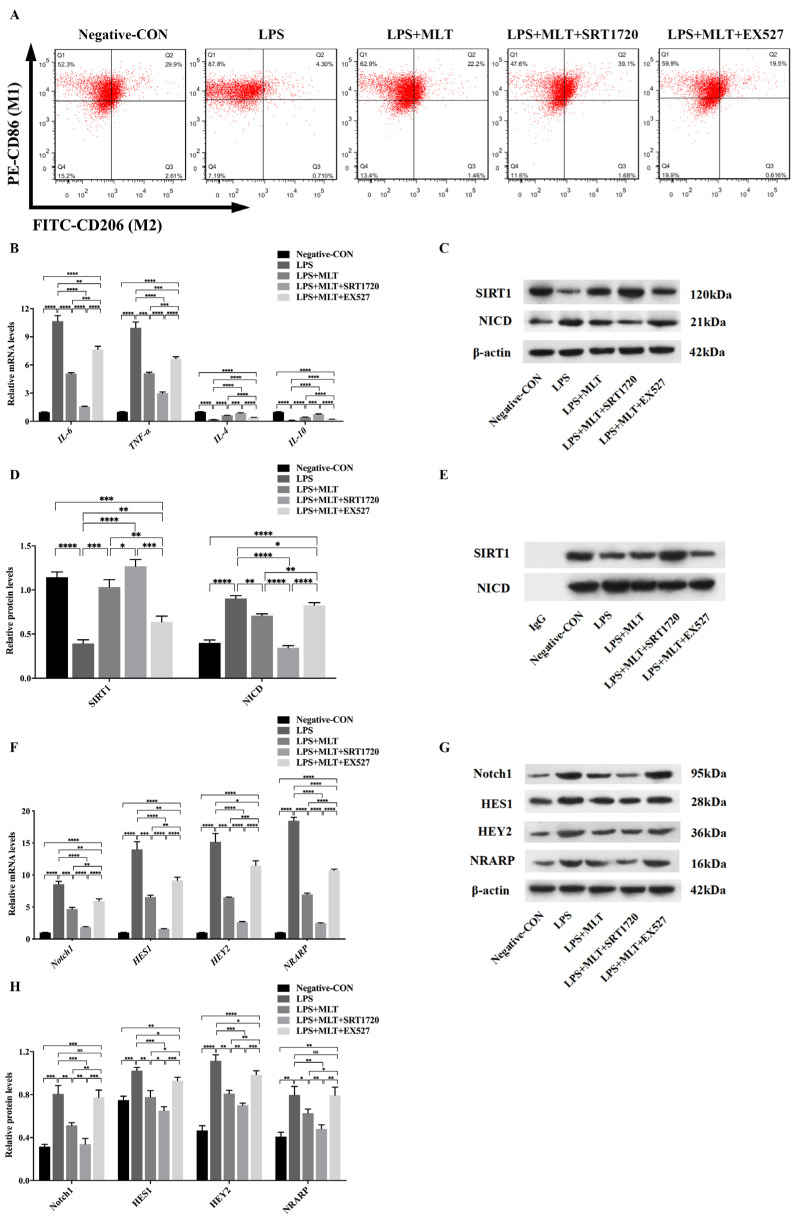
Regulation of SIRT1/Notch signal pathway contributes to the effect of MLT on inhibiting M1-type Mφ polarization. RAW 264.7 Mφs were exposed to LPS for 6 h and followed by MLT treatment for 24 h, then treated with SIRT1 agonists (SRT1720) or inhibitors (EX527) for another 6 h and followed by different analysis. (**A**) Reduction of the ratio of M1-type Mφs while enhancement of the ratio of M2-type Mφs after the treatment with MLT, aggravated in combination with SRT1720, and reversed in the presence of EX527, was determined by flow cytometry assay. (**B**) The relative mRNA levels of M1-type Mφ genes (*IL-6* and *TNF-α*) and M2-type Mφ genes (*IL-4* and *IL-10*) were measured by RT-qPCR analyses (*n* = 3). The levels of *IL-6* and *TNF-α* mRNA were reduced in the presence of MLT treatment, downregulated in combination with SRT1720, and upregulated in the presence of EX527. (**C**,**D**) The relative protein levels of SIRT1 and NICD in Mφs were measured by Western blot analyses (*n* = 3). (**C**) Western blotting showed the upregulation of the SIRT1 protein with the downregulation of the NICD protein, aggravated in combination with SRT1720, and reversed in the presence of EX527. (**D**) Quantitative analysis showed the increased level of the SIRT1 protein, with the reduced level of the NICD protein after the treatment with MLT, aggravated in combination with SRT1720, and reversed in the presence of EX527. (**E**) IP with the anti-NICD antibody was used to verify the interaction between SIRT1 and NICD. The interaction between SIRT1 and NICD in the LPS + MLT-treated Mφs was markedly increased compared with that in the LPS-treated Mφs. Moreover, treatment with SRT1720 further aggravated this interaction, while treatment with EX527 ameliorated the interaction. (**F**) The relative mRNA levels of downstream target genes of the SIRT1/Notch signaling pathway were measured by RT-qPCR analyses in Mφs (*n* = 3). The levels of *Notch1*, *HES1*, *HEY2* and *NRARP* mRNA were downregulated after the treatment with MLT, downregulated in combination with SRT1720, and upregulated in the presence of EX527. (**G**,**H**) The relative protein levels of downstream factors of the SIRT1/Notch signaling pathway were measured by Western blot analyses in Mφs (*n* = 3). (**G**) Western blotting showed the downregulation of downstream factors protein of the SIRT1/Notch signaling pathway in Mφs. (**H**) Quantitative analysis showed the reduced levels of Notch1, HES1, HEY2 and NRARP protein after the treatment with MLT, downregulated in combination with SRT1720, and upregulated in the presence of EX527. Data are expressed as mean ± standard deviation. The two groups among the five groups are compared by using an independent *t*-test. * *p* < 0.05, ** *p* < 0.01, *** *p* < 0.001, **** *p* < 0.0001.

Since SIRT1 has been identified as an important regulatory factor of the inflammatory response, whether MLT inhibits M1-type polarization of Mφs via the SIRT1 pathway was further explored. RAW 264.7 Mφs were exposed to LPS (1 μg/mL) for 6 h and followed by MLT treatment for 24 h, then treated with SIRT1 agonists (SRT1720) or inhibitors (EX527) for another 6 h. Western blot showed that the level of SIRT1 was dramatically decreased in LPS-treated Mφs when compared with those in the negative control Mφs, and the further addition of MLT treatment to LPS-treated Mφs increased the expression of SIRT1 when compared with the LPS-treated Mφs. SIRT1 level significantly increased in the LPS + MLT + SRT1720-treated Mφs when compared with that in the LPS + MLT-treated group. While the reversal change of SIRT1 level was achieved in the LPS + MLT + EX527-treated Mφs when compared with that in LPS + MLT-treated Mφs (Figure 5C,D). These data indicated that SIRT1 was involved in the effect of MLT on inhibiting M1-type polarization of Mφs, and this effect could be partially attenuated by the EX527 treatment. In comparison with the change of SIRT1 level, acetylated NICD expression showed the opposite trend in each group (Figure 5C,D). These results revealed that SIRT1 activation was negatively associated with the NICD levels. As shown in Figure 5A, the flow cytometry results showed that SRT1720 markedly inhibited M1-type polarization of Mφs, while EX527 promoted M1-type polarization of Mφs. Moreover, the levels of M2-type Mφ markers like IL-4 and IL-10 were markedly upregulated, while the levels of M1-type Mφ markers IL-6 and TNF-α were significantly downregulated in the LPS + MLT + SRT1720-treated Mφs, relative to those in the LPS + MLT-treated group (Figure 5B). In contrast, the levels of M1-type Mφ markers, including IL-6 and TNF-α were all increased, while the levels of M2-type Mφ markers, including IL-4 and IL-10, were markedly declined in LPS + MLT + EX527-treated Mφs when compared with those in the LPS + MLT-treated Mφs (Figure 5B). Thus, the data suggested that SIRT1 participated in regulation of M1-type polarization of Mφs. More specifically, the downregulation of SIRT1 could promote the M1-type polarization of Mφs, while the upregulation of SIRT1 could inhibit this process.

To explore the mechanism of MLT in the regulation of M1-type polarization of Mφs, IP with anti-NICD antibody was used to verify the interaction between SIRT1 and NICD. As shown in Figure 5E, the interaction between SIRT1 and NICD in the LPS + MLT-treated Mφs was markedly increased compared with that in the LPS-treated Mφs. Moreover, treatment with SRT1720 further aggravated this interaction, while treatment with EX527 ameliorated the interaction. In addition, both RT-qPCR and Western blot revealed that the expression of Notch1 was significantly decreased in the LPS + MLT-treated Mφs relative to that in the LPS-treated Mφs. Further treatment with SRT1720 downregulated the level of Notch1, whereas treatment with EX527 upregulated the expression of Notch1 (Figure 5F–H). The results indicated that the SIRT1/Notch signal pathway maybe partially contributes to the effect of MLT on inhibiting M1-type polarization of Mφs; its specific mechanism might be that MLT could activate SIRT1, which further inhibits Notch signaling through NICD deacetylation. To confirm the hypothesis, the expression levels of downstream target genes of the SIRT1/Notch signaling pathway were explored. The RT-qPCR results showed that levels of genes, such as *HES1*, *HEY2* and *NRARP*, were significantly upregulated in the LPS-treated Mφs, which was inhibited by MLT and was further inhibited by MLT + SRT1720 via upregulation of SIRT1 levels (Figure 5F). In addition, the inhibition effect by MLT was partly attenuated by downregulation of SIRT1 levels after using EX527 (Figure 5F). Consistent with RT-qPCR results, Western blotting further demonstrated that protein levels of the above factors showed similar trends (Figure 5G,H). These data suggested that MLT could inhibit M1-type polarization of Mφs, partially through upregulating SIRT1 expression and downregulating Notch expression.

### 3.6. Evaluation of Therapeutic Effect of MLT on Rat Model of IDD

**Figure 6:**
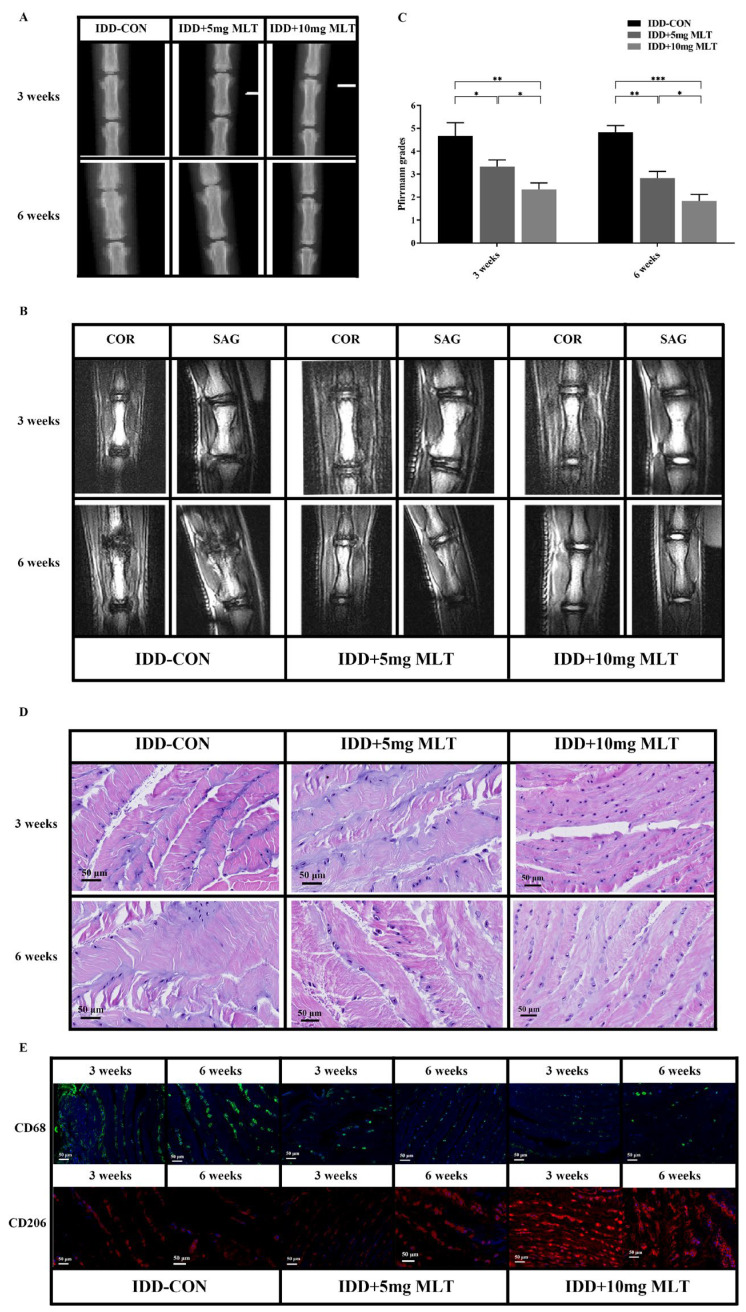
Inhibition of M1-type Mφ polarization by MLT ameliorates IDD in vivo. Rat models of IDD were established by caudal IVD puncture surgery and with the treatment of MLT for 1 week, then followed by different analysis at 3 weeks and 6 weeks after surgery. (**A**) The representative X-ray radiographs showed the different heights of IVDs in different groups at 3 weeks and 6 weeks after surgery. The height of IVDs in the IDD control group was lower than that in the MLT-treated groups both at 3 and 6 weeks. (**B**) The representative coronal (COR) and sagittal (SAG) MRI of every group at 3 weeks and 6 weeks after surgery. The MLT-treated groups had higher IVD height and T2-weighted signal intensity when compared with the IDD control group at both 3 and 6 weeks. (**C**) The Pfirrmann grade scores of the IVDs in each group assessed by the results of T2-weighted MRI to quantify the degeneration degree of IVDs (*n* = 3). The Pfirrmann grades were significantly lower in the MLT-treated groups relative to those in the IDD control group at both 3 and 6 weeks. (**D**) H&E staining of IVD tissues at 3 weeks and 6 weeks after surgery showed the degrees of degeneration in IVDs in different groups. The NP cells showed reduced size and number and were surrounded by disorganized AF, and the cells of IVDs were clustered and the striped matrices were distributed among the cell clusters in the IDD-control group, while the morphological changes of NP cells and the degrees of the firosis and structural disorder of IVDs were markedly ameliorated in the MLT-treated groups. (**E**) The condition of Mφ polarization in IVD tissues at 3 weeks and 6 weeks after surgery were measured by immunofluorescence. M1-type Mφs (CD68+, green) decreased, while M2-type Mφs (CD206+, red) increased in the MLT-treated groups when compared with the IDD-control group, both at 3 and 6 weeks. Data are expressed as mean ± standard deviation. The two groups among the three groups are compared by using an independent *t*-test. * *p* < 0.05, ** *p* < 0.01, *** *p* < 0.001.

To explore the medical potential of MLT on degenerative IVDs in vivo, the rat IDD model was established and treated with MLT, and then the effects assessed of MLT on the IDD process in rats. X-ray radiograph, MRI and evaluation of Pfirrmann grade scores were taken at 3 and 6 weeks after surgery. The X-ray data showed that the height of IVDs in the IDD-control group was lower than that in the MLT-treated groups (5 mg/kg-MLT and 10 mg/kg-MLT-treated) both at 3 and 6 weeks (Figure 6A). The MRI results showed that the MLT-treated groups had higher IVD height and T2-weighted signal intensity when compared with the IDD-control group both at 3 and 6 weeks, and this manifestation was more obvious in the 10 mg MLT-treated group (Figure 6B). In addition, the Pfirrmann grades, which were based on T2-weighted images of MRI and manifested the degree of IVD degeneration, were significantly lower in the MLT-treated groups relative to those in the IDD-control group both at 3 and 6 weeks (Figure 6C). 

Furthermore, the data of H&E staining and immunofluorescence further demonstrated the therapeutic effect of MLT. As shown in H&E staining, the NP cells in the IDD control group showed reduced size and number and were surrounded by disorganized AF. The cells of degenerative IVDs were clustered and the striped matrices were distributed among the cell clusters, demonstrating the bad degeneration of NP cells. In comparison with the IDD control group, the morphological changes of NP cells and the degrees of the firosis and structural disorder of degenerative IVDs were markedly ameliorated in the MLT-treated groups (Figure 6D). The immunofluorescence results showed the condition of Mφ polarization in rat IVD tissue. Compared with the IDD control group, M1-type Mφs (CD68+) decreased, while M2-type Mφs (CD206+) increased in the MLT-treated groups both at 3 and 6 weeks (Figure 6E). These results demonstrated that MLT could inhibit M1-type polarization of Mφs and ameliorate IDD in rats in vivo.

## 4. Discussion

Inflammatory response has been acknowledged as playing a pivotal role in the pathogenesis of IDD. IDD is characterized by the upregulation of pro-inflammation-related cytokines in IVD cells, which not only directly disrupts disc matrix homeostasis, but also indirectly affects matrix homeostasis by promoting the infiltration and activation of immune cells, especially macrophages, therefore amplifying the inflammatory cascade [29,30]. Pro-inflammatory M1-type Mφ has been identified as the main type of immune cell and an important inflammatory regulator by secreting a variety of cytokines, which are implicated in the pathogenesis of IDD [6]. In this study, we found that LPS stimulation could promote M1-type polarization of Mφs as shown by a higher M1/M2 ratio, and increased mRNA and protein expression levels of M1-type Mφ markers including IL-6 and TNF-α, and decreased M2-type Mφ markers, including IL-4 and IL-10. In addition, M1-type Mφs could induce molecular changes associated with IDD in NP cells, including increased expression of pro-inflammation-related cytokines (IL-6, TNF-α, iNOS and COX-2), oxidative stress response (the levels of MDA and ROS) and matrix degradation markers (MMP-13, ADAMTS4 and ADAMTS5) and reduced matrix anabolic markers (COL2A1 and TIMP-1). In vitro experiments performed by Yamamoto et al. showed that the production of pro-inflammatory cytokines in the co-culture of human primary NP cells and Mφs was significantly higher than those in the independent culture group [31]. Yang et al. investigated the effects of secreted factors in the conditioned media of rat NP cells on mouse RAW 264.7 Mφs and vice versa demonstrated that the biological interactions between infiltrating Mφs and NP cells lead to increasingly severe inflammatory conditions, which further promoted IDD development [7]. These studies, together with this current study, indicate that LPS treatment could promote M1-type polarization of Mφ and M1-type Mφs could further induce NP cell injury, which might serve as a potential mechanism to find a new therapeutic method to ameliorate the process of IDD. 

MLT (N-acetyl-5-methoxytryptamine) is an indoleamine hormone mainly produced and secreted by the pineal gland and is widely known as a regulator of circadian rhythms [32]. Recently, increasing evidence has shown that MLT has diverse biological functions, including antioxidant [33,34,35,36], anti-inflammatory [37,38,39], anti-apoptosis [40,41] and immunomodulation [42,43,44]. Our previous study has discovered that MLT could promote proliferation, induce autophagy, and suppress apoptosis on annulus fibrosus cells, implying a role of melatonin in alleviating IDD. The potential role of MLT in IDD has also been revealed by other researchers. In vivo studies have shown that exogenous MLT administration activated the recovery process in the degenerative IVD tissue in rats [45,46], whereas surgical pinealectomy in chicken significantly reduced serum melatonin levels as well as accelerating the deterioration of IDD [14]. Moreover, in vitro studies have confirmed the existence of MT1 and MT2 in IVD tissues (especially, in NP cells) and the ROR in Mφs. MLT could combine with these receptors to enhance the proliferation of NP cells in a dose-dependent manner, and inhibit M1-type Mφ polarization and expression levels of pro-inflammation-related genes, thus playing a vital role in the prevention of IDD [47,48]. In this study, in vitro experiment results demonstrated that MLT treatment could alleviate the M1-type Mφ polarization and reduce the levels of mRNA and protein of pro-inflammatory cytokines in RAW 264.7 Mφs, thus reducing the NP cell injury. Moreover, in vivo study clarified that MLT could inhibit M1-type polarization of Mφs and ameliorate the degree of degenerative IVDs in rat models of IDD in vivo. Although oral melatonin is mostly perceived as safe based on published reports, the contraindications and potential side effects of MLT still need to be considered. Caution should be exercised when MLT is taken with one or more drugs; otherwise, side effects (like extreme sedation) will appear. For instance, one of the famous interactions is melatonin with the anti-depressant medication, fluvoxamine. It can result in the potentiation of melatonin levels due to reducing its degradation. Therefore, patients diagnosed with any disease needing MLT should consult their health professionals and take it under medical supervision [49].

In addition, SIRT1 is a secondary mediator of the anti-inflammation effects of MLT. SIRT1 has also been shown to exert its anti-inflammation action by inhibiting Notch signaling. Therefore, the beneficial effect of MLT in improving IDD could be derived from the activation of the SIRT1/Notch signaling pathway and then inhibiting inflammatory reactions. According to our findings, MLT treatment could dramatically upregulate SIRT1 levels and downregulate acetylated NICD levels and certain Notch-related markers including Notch1, HES1, HEY2 and NRARP in M1-type Mφs. The IP data showed that the interaction between SIRT1 and NICD was also increased after the melatonin treatment in M1-type Mφs. Moreover, these changes of the SIRT1/Notch signaling pathway were partly reversed in the exposure of MLT-treated M1-type Mφs to inhibitor EX527, while they were further promoted in the exposure to agonist SRT1720. These results demonstrated that SIRT1 could negatively regulate the Notch signaling pathway, which mediated the beneficial effects of MLT in IDD. 

Of course, there were certain limitations in this research. First of all, the study primarily relied on in vitro and animal models, which may not fully reflect the complex physiological conditions in humans. Further study will be conducted to explore the medical potential of MLT in human IDD patients. In addition, this study preliminary demonstrated the SIRT1/Notch signaling pathway played a role in the influence of MLT on IDD, but a more detailed mechanism study was not included. Subsequent studies on this part may be helpful to understand the precise molecular interactions and downstream effects.

In conclusion, current studies demonstrate that MLT could ameliorate NP cell injury caused by inflammation in vitro and vivo, partially through inhibiting M1-type polarization of Mφs via regulating the SIRT1/Notch signaling pathway. It is of great significance for the remission of IDD. SIRT1 could be regarded as a secondary mediator of the anti-inflammatory effects of MLT, and SIRT1-mediated deacetylation of the NICD led to NICD-degradation and Notch signaling inhibition, which resulted in the inhibition of M1-type Mφ polarization. The findings provide novel evidence revealing the Mφ polarization involved in IDD and the possible signaling pathway by which MLT targets Mφ polarization, demonstrating MLT treatment as a promising therapeutic method for the management for IDD.

## Figures and Tables

**Table 1:** The list of primers was utilized for RT-qPCR.

Gene	Forward Primer (5′-→-3′)	Reverse Primer (5′-→-3′)
*IL-4*	CTCGAATGTACCAGGAGCCA	AGTCTCTGCAGCTCCATGAG
*IL-6*	ATACCACTCCCAACAGACCT	GTTCTTCATGTACTCCAGGT
*IL-10*	TAACTGCACCCACTTCCCAG	GTCTTCAGCTTCTCACCCAG
*TNF-a*	CCACGCTCTTCTGTCTACTG	CCTTGAAGAGAACCTGGGAG
*iNOS*	CTGGACAAGCTGCATGTGAC	TGGGTCCTCTGGTCAAACTC
*COX-2*	CATAAGCGAGGACCTGGGTT	TGGCATACATCATCAGACCA
*Notch1*	TAAGGACCTCAAGGCACGGA	CATCTGACAAGTAGCCATG
*HES1*	GATCAACGCCATGACCTACC	GTTGGGGATGAGAAAGGCAA
*HEY2*	GAGCATTGGATTCCGAGAGT	GGGTTGACTCTGATGTGTGG
*NRARP*	CAGCACTACACCAGTCAGTC	ACTTGGCCTTGGTGATGAGA
*COL2A1*	TGAAGGGTGAGAGTGGTTCC	ACGAGAACCTTGAGCACCTT
*TIMP-1*	AGACCACCTTATACCAGCGT	GAGAACCTTGAGCACCTTCA
*MMP-13*	TCTATGATGGCACTGCTGAC	GTTGTAGCCTTTGGAACTG
*ADAMTS4*	CATCACTGACTTCCTGGACA	CGAAGGTCAGTTGGCATTG
*ADAMTS5*	GGCAGACGTTGGGACCATAT	TCTGTGATGGTGGCTGACGT

## Data Availability

The datasets generated and analyzed during the current study are available from the corresponding authors upon reasonable request.
